# Evaluation of antioxidant property of heat shock protein 90 from duck muscle

**DOI:** 10.5713/ajas.19.0854

**Published:** 2020-05-12

**Authors:** Muhan Zhang, Daoying Wang, Xinglian Xu, Weimin Xu

**Affiliations:** 1 Key Lab of Meat Processing and Quality Control, Ministry of Education, Nanjing Agricultural University, Nanjing 210095, China; 2 Institute of Agricultural Products Processing, Jiangsu Academy of Agricultural Sciences, Nanjing 210014, China

**Keywords:** 90 Kda heat shock protein (Hsp90), Oxidized Phospholipids, Radical, Electron Paramagnetic Resonance (EPR)

## Abstract

**Objective:**

The objectives of this study were to investigate the direct antioxidative effect of 90 Kda heat shock protein (Hsp90) obtained from duck muscle.

**Methods:**

The interaction of Hsp90 with phospholipids and oxidized phospholipids was studied with surface plasmon resonance (SPR), and their further oxidation in the presence of Hsp90 was evaluated with thiobarbituric acid reactive substances (TBARS) assay. The scavenging effect on the 1,1-diphenyl-2-picrylhydrazyl (DPPH) and 2,2′-azinobis (3-ethylbenzthiazoline-6-sulfonic acid (ABTS) was measured, and the electron paramagnetic resonance (EPR) spectroscopy in combination with 5-tert-Butoxycarbonyl-5-methyl-1-pyrroline-N-oxide and 2-phenyl-4,4,5,5,-tetramethylimidazoline-1-oxyl-3-oxide (PTIO) was utilized to determine the abilities of Hsp90 in scavenging hydroxyl and PTIO radicals.

**Results:**

SPR showed Hsp90 could bind with both phospholipids and oxidized phospholipids, and prevent their further oxidation by the TBARS assay. The DPPH and ABTS scavenging activity increased with Hsp90 concentration, and could reach 27% and 20% respectively at the protein concentration of 50 μM. The EPR spectra demonstrated Hsp90 could directly scavenge ·OH and PTIO· radicals.

**Conclusion:**

This suggests that Hsp90, a natural antioxidant in meat, may play an important role in cellular defense against oxidative stress, and may have potential use in meat products.

## INTRODUCTION

Oxidative stress and resulting lipid peroxidation is involved in numerous pathological states including inflammation, atherosclerosis, hypertension, and cancer. Reactive oxygen species (ROS) and reactive nitrogen species (RNS) such as hydroxyl radical (·OH), superoxide anion (O_2_^−^), hydrogen peroxide (H_2_O_2_), and iron-oxygen complexes are highly reactive to initiate lipid peroxidation and are known to have deleterious effects in eukaryotes [1]. And compared to free radicals, due to the longer half-life and the ability to diffuse from their site of formation, the breakdown products of lipid peroxides may serve as ‘oxidative stress second messengers’ [2]. Those breakdown products of lipid peroxidation, mostly aldehydes, such as malonaldehyde (MDA), hexanal, and 4-hydroxynonenal (HNE) have received a lot of attention because they are biologically active compounds and have been considered as toxic end-products [2]. Polyunsaturated fatty acids of phospholipid are the most common substrates of oxidative attack, leading to the generation of lipid hydroperoxides, which are unstable and can undergo carbon-carbon bond cleavage giving rise to the formation of short chain aldehydes and other end-products [3]. The phospholipid oxidation products promote the formation of ROS in vascular endothelial cells, induce apoptosis in vascular smooth muscle cells, and play dominant roles in chronic inflammatory processes [4]. In rabbit meat the MDA was reported to promote the generation of ROS and enhance the protein oxidation [5].

Once the redox balance is disrupted, a number of proteins and peptides could function as antioxidants to protect cells and organisms from oxidative damage. Heat shock proteins (Hsps) are a family of proteins ubiquitously expressed under stress conditions as well as under physiological conditions. The 90 Kda heat shock protein (Hsp90) is an important member of Hsps family that have versatile functions in regulating cellular homeostasis and promoting cell survival. It is abundantly expressed in cells and tissues that accounts for 1% to 2% of total proteins under non-stressed conditions and is upregulated to the level of 4% to 6% of cellular proteins in response to stress [6]. Hsp90 could play important roles in cell survival and is implicated as an inhibitor of programmed cell death during oxidative stress [7]. It was protective against oxygen free radical damage induced apoptosis in human gastric mucosal cells [8]. Hsp90 has also been identified to be an iron binding protein associated with the membrane of Hela cells [9]. However, there is no work on the direct antioxidative ability of Hsp90.

Our previous study demonstrated that Hsp90 isolated from muscle could inhibit phospholipid oxidation and confer tolerance to the transformed *Escherichia coli* (*E. coli*) cells containing *Hsp90* gene exposed to H_2_O_2_ treatment [10]. It is possible that the putative antioxidant effect of Hsp90 may be associated with the ability to bind oxidized phospholipids, and Hsp90 thus serving as a scavenger of oxidation products. Lipid oxidation could lead to rancid odour, off flavor development, discolouration, loss of nutritional values and generation of harmful substances in meat. The presence of Hsp90 may enhance the antioxidant potential of muscle and may be useful as a natural antioxidant. The aim of the present studies was to examine the binding ability of Hsp90 to oxidized phospholipids and the radical scavenging ability of Hsp90.

## MATERIALS AND METHODS

### Purification of Hsp90

Hsp90 was extracted and purified from duck muscles according to our previous study [10]. Thirty g of muscle sample was homogenized with 100 mL Tris-HCl buffer (100 mM, pH 8.0) at 12,000 rpm using an Ultra Turrax (T25, IKA, Staufen, Germany). The homogenate was then centrifuged for 20 min at 12,000 g (Allegra 64R, Beckman, Brea, CA, USA), supernatant was collected and filtered through four-layer gauze. Afterwards, the filtrate was precipitated by ammonium sulphate at a saturation of 70% at 4°C for 12 h under gentle stirring, and centrifuged for 20 min at 12,000 g, the precipitate was dissolved in Tris-HCl buffer (100 mM, pH 8.0) and dialyzed for 24 h in Tris-HCl buffer (100 mM, pH 8.0).

The crude protein was loaded in a DEAE-cellulose 52 column (2.5 cm×60 cm) with Tris-HCl buffer (50 mM, pH 8.0), and the target protein peak was eluted with Tris-HCl buffer (50 mM, pH 8.0) containing 1 M NaCl. Then the fraction containing Hsp90 was applied to Source 15Q column (1.0 cm×5.0 cm), which was previously equilibrated with Tris-HCl buffer (50 mM, pH 8.0) and eluted with the same buffer, until unbound protein had passed through the column. The bound proteins were eluted with a linear gradient of 0 to 1.0 M NaCl in equilibration buffer. Hsp90 was pulled and concentrated by ultrafiltration using a 10 kDa cut-off membrane. The protein concentration was determined with the Bradford Protein Assay Kit (Jiancheng, Nanjing, China), and the purity of protein was analyzed by sodium dodecyl sulfate-polyacrylamide gel electrophoresis.

### Preparation of liposomes from phospholipids

Liposome vesicles were prepared by dissolving a defined amount of L-a-1-palmitoyl-2-arachidonoyl-sn-glycero-3-phosphorylcholine (PAPC) in chloroform in a round bottom flask, followed by drying under a rotary evaporator and the lipid film was left under vacuum overnight to remove all traces of solvent. Phosphate-buffered saline (PBS) buffer (pH 7.0) was added to the lipid and the dispersion was vortexed. Subsequently the liposome suspension was sonicated in a bath sonicator at 4°C until a clear suspension of unilamellar vesicles was obtained. The vesicles were then extruded through 100 nm polycarbonate membranes mounted on an extruder for 20 times (Morgec Inc., Shanghai, China). The oxidized PAPC (oxPAPC) liposome was generated by incubating PAPC liposome with H_2_O_2_ at 37°C for 4 h.

### Surface plasmon resonance measurements

SPR experiments were conducted on a Biacore X100 Plus (GE Healthcare, Uppsala, Sweden) equipped with L1 chip (GE Healthcare, Sweden). Before each liposome injection, the chip surface was cleaned by injecting 91 μL of 40 mM octylglucoside, and washed thoroughly with PBS buffer. Liposomes (0.5 mg/mL) were deposited on sensor chips at a low flow rate (10 μL/min) until a stable resonance unit level was obtained, then it was washed with 2×50 mM NaOH to remove unstably bound liposome. To block the liposome-unpacked surface, the chip surface was treated with 0.1 mg/mL BSA for 1 min at 10 μL/min. Hsp90 solutions in PBS buffer were injected at various concentrations from 0.14 to 2.22 μM, and the lipid-coated surface was regenerated with 25 mM NaOH as long as a consistent baseline was maintained. All analyses were performed at 25°C.

### Thiobarbituric acid reactive substances

The PAPC and oxidized PAPC liposome with or without Hsp90 (lipid:protein = 100:1, w/w) was incubated at 37°C for 1h respectively, and an aliquot of sample was mixed with 1 mL of TBA/TCA solution (15 mM TBA/15% trichloroacetic acid [TCA; W/V]) and incubated in a boiling water bath for 15 min. After cooling, the mixture was centrifuged at 15,000×g for 10 min. The absorbance of the supernatant was determined at 532 nm against a reagent blank. The level of lipid oxidation in the liposome solution was expressed as 2-thiobarbituric acid reactive substances (TBARS) value (mmol MDA/mg phospholipid) calculated using the molar extinction coefficient of 1.56×10^5^ M^−1^ cm^−1^.

### 1, 1-Diphenyl-2-picrylhydrazyl radical scavenging activity assay

Different concentrations of Hsp90 was mixed with 300 μL 0.5 mM 1, 1-diphenyl-2-picrylhydrazyl (DPPH) solution. After standing at 37°C for 30 min, the absorbance of the mixture at 517 nm was measured. Deionized water was used as a control and glutathione (GSH) was used as a positive control. The activity is mentioned in percentage form of DPPH radical scavenging according to following equation:

Scavenging activity (%)=[(Absorbance of control-Absorbance of test)/Absorbance of control]×100

### 2,2′-Azinobis(3-ethylbenzthiazoline-6-sulfonic acid radical scavenging activity assay

The 2,2′-azinobis(3-ethylbenzthiazoline-6-sulfonic acid (ABTS) stock solution was produced by the reaction of 7 mM of ABTS solution with 2.45 mM potassium persulfate. The mixture was kept in the dark at room temperature for 16 h before use. The stock solution was diluted with distilled water and equilibrated at room temperature to give an absorbance at 0.70±0.05 at 734 nm in a cuvette. Then, 10 μL of each sample with different concentrations of Hsp90 were added to 300 μL ABTS working solution and vortexed for 30 s. Deionized water was used as a control and GSH was used as a positive control. The absorbance at 734 nm was measured according to the equation:

Scavenging activity (%)=[(Absorbance of control-Absorbance of test)/Absorbance of control]×100

### Electron paramagnetic resonance spectroscopy

The electron paramagnetic resonance (EPR) measurements were made at room temperature with a Bruker EMX-10/12 EPR spectrophotometer (Bruker, Rheinsteten, Germany). EPR parameters were 3480 G Field set, 200 G sweep width, 1.0 G modulation amplitude, and 20 mW microwave power. The reaction generating ·OH was initiated by adding FeCl_2_ and H_2_O_2_ in sodium phosphate buffer (50 mM, pH 7.4). For spin trapping of ·OH, the final concentration of 5-tert-Butoxycarbonyl-5-methyl-1-pyrroline-N-oxide (BMPO) was adjusted to 20 mM. EPR signal of 2-phenyl-4,4,5,5,-tetramethylimidazoline-1-oxyl-3-oxide (PTIO·) was detected with 2-(4-Carboxyphenyl)-4,4,5,5,-tetramethylimidazoline-1-oxyl-3-oxide (carboxy-PTIO), which was prepared at the final concentration of 0.5 mM. The Hsp90 was mixed with ·OH or PTIO· generating solution at the final concentration of 2.5 μM.

### Statistical analysis

All data were expressed as the mean±standard deviation of at least three replicates. Statistical analysis of the differences between each group was evaluated by one-way analysis of variance using the SPSS 18.0. Duncan’s multiple comparison was used to compare the differences among groups. Values of p<0.05 was considered as statistically significant.

## RESULTS AND DISCUSSION

### Binding of Hsp90 with oxidized phospholipid and prevention of its oxidation

**Figure 1:**
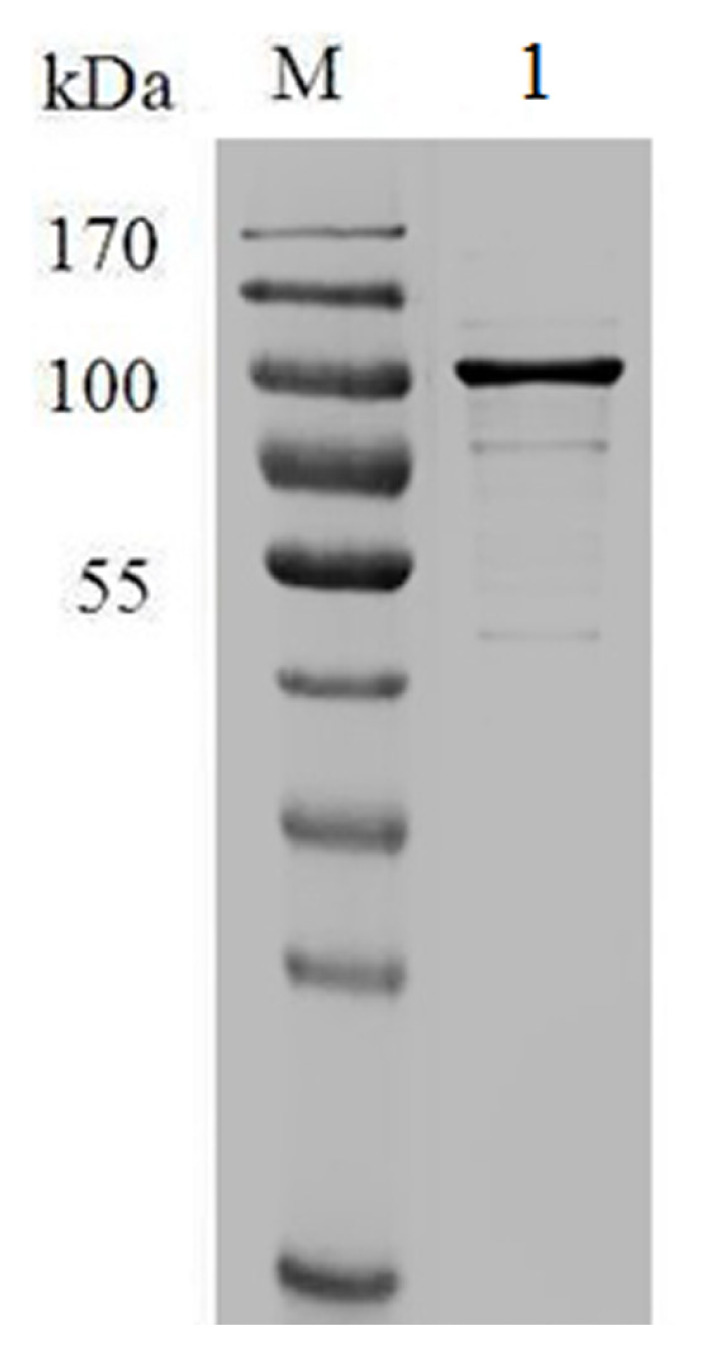
Verification of Hsp90 with sodium dodecyl sulfate-polyacrylamide gel electrophoresis. Hsp90, 90 Kda heat shock protein. Lane M-molecular weight marker, Lane 1-purified Hsp90.

**Figure 2:**
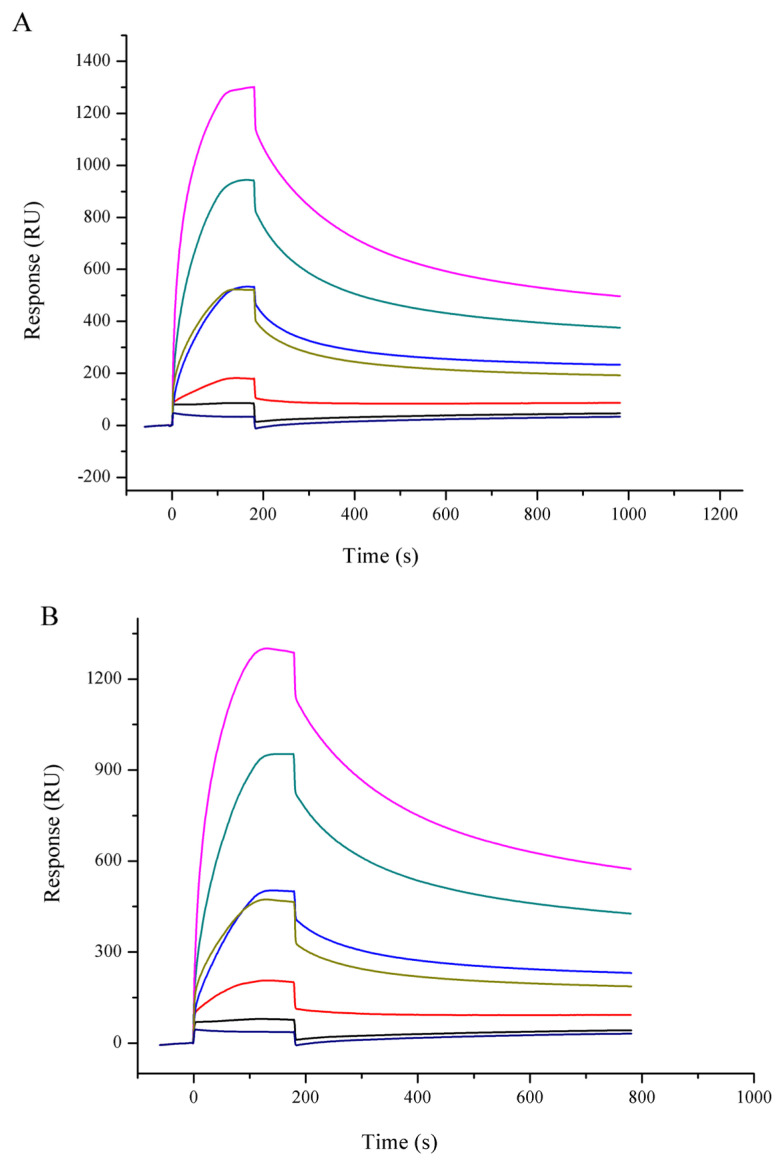
Binding sensorgram of Hsp90 with PAPC (A) and PAPC hydroperoxide (B) by SPR. A series concentration of Hsp90 (0.14 to 2.22 μM) were injected on the chip loaded with liposome. Hsp90 rapidly attained equilibrium binding and showed dose-dependent responses. Hsp90, 90 Kda heat shock protein; PAPC, L-a-1-palmitoyl-2-arachidonoyl-sn-glycero-3-phosphorylcholine; SPR, surface plasmon resonance.

Hsp90 was extracted from muscle with high purity (Figure 1). The sensorgrams for the interaction of Hsp90 with lipids are shown in Figure 2, and the kinetic parameters are presented in Table 1. The equilibrium constants for dissociation (KD) was lower with PAPC, but it was not significantly different (p>0.05), showing that Hsp90 had slightly higher affinity to PAPC. However, the Hsp90 binding level (Rmax) was significantly higher with PAPC hydroperoxides (p<0.05).

**Figure 3:**
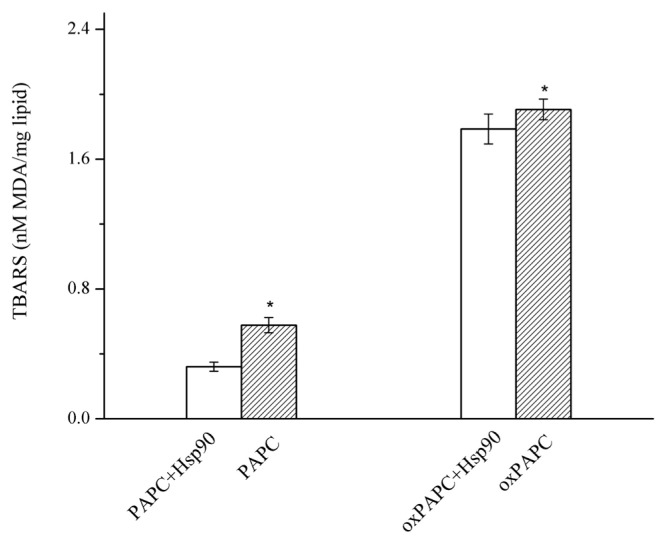
Effect of Hsp90 added to PAPC and oxidized PAPC liposomes on formation of MDA. Lipid alone had significantly higher MDA levels than the lipid added with Hsp90 (p<0.05). Hsp90, 90 Kda heat shock protein; PAPC, L-a-1-palmitoyl-2-arachidonoyl-sn-glycero-3-phosphorylcholine; MDA, malonaldehyde; oxPAPC, oxidized PAPC. Bars indicate standard deviation. * Indicates significant difference between lipid alone (PAPC or oxPAPC) and lipid added with Hsp90 (PAPC+ Hsp90 or oxPAPC+Hsp90) (p<0.05).

The PAPC and PAPC hydroperoxides were oxidized at 37°C, and MDA, one of the oxidation end products, was measured as an indicator of lipid oxidation. As shown in Figure 3, the lipid alone had significantly higher MDA levels than the lipid with added with Hsp90 (p<0.05), indicating Hsp90 prevented PAPC and oxPAPC from further oxidation.

Lipid peroxidation is induced from enhanced generation of ROS or reduced antioxidant defense, which are involved in pathological conditions or under conditions of oxidative stress. Cell membranes and intramuscular lipids of meat are enriched in phospholipids containing polyunsaturated fatty acids, which are highly prone to oxidative attack. Lipid hydroperoxides generated from phospholipids and unsaturated fatty acids are the intermediates of peroxidation reactions, and can be further oxidized to form hundreds of short-chain secondary products [3]. HNE and MDA could be produced from 13-hydroperoxyl linoleoyl or 15-hydroperoxy arachidonic acid [11], or could be rapidly generated from the decomposition of phospholipid hydroperoxides [12]. The accumulation of these secondary products such as MDA and HNE could influence the ROS generating system and facilitate protein oxidation, they also have cytotoxicity which could induce DNA fragmentation or protein modification and finally contribute to disease progression [2,5]. Hsp90, known as stress protein, is triggered by oxidative stress and other adverse conditions, and confers protection to cells and tissues against oxidative stress along with other Hsps. A number of studies have shown oxidative stress can alter the fatty acid composition of membranes and result in cell injury due to loss of membrane integrity, whereas small Hsps and Hsp70 could bind with phospholipid, preserve the integrity of membranes, and involved in the defense system during oxidative stress [13–15]. Hsp90 and Hsp70 has also been reported to bind with hydroxyeicosatetraenoic acid, an oxidation product of arachidonic acid, which play a role in the pathogenesis of some diseases [16,17]. In our previous study, the property of Hsp90 in the binding of phospholipids, regulation of membrane physical state and protection of *E. coli* cells against oxidation was found [10,18]. The short chain end products are highly reactive to initiate the lipid oxidation, and they are also sources that can induce Hsps production, the expressed Hsps may in turn function as the antioxidant to reduce the oxidation of phospholipids and oxidized phospholipids (OxPLs), and reduce the accumulation of these end products.

Oxidized phospholipids have been shown to accumulate at the sites of chronic inflammation and oxidative tissue damage, where they not only affect biophysical properties of membranes, but also can be considered a potential danger that modulates immune responses [3]. There are indications that OxPLs, but not its unoxidized precursor, may increase intracellular and extracellular superoxide levels in human and bovine aortic endothelial cells [19]. Binding to oxPLs by proteins and peptides could facilitate their sequestration/metabolism/clearance in the body and reduce the inhibitory effect of oxPLs on various antioxidant enzymes associated with the lipoproteins and cell membranes [20]. Some proteins such as human serum paraoxonases (PON-1) protect against oxidation by augmenting the hydrolysis of oxidized lipids and decompose hydroperoxides [21]. The binding of Hsp90 to phospholipids and oxidized phospholipids might not only protect them from further oxidation to produce a variety of biological and pathophysiological substances, but also facilitate the metabolism and clearance of the OxPLs. Some antioxidant could quench the chain reaction and prevent oxidation of lipids, but it does not alter the lipid hydroperoxides that are already formed, while Hsp90 has the ability to bind with OxPLs to prevent their further oxidation to more reactive and pathogenic end products.

### DPPH and ABTS radical scavenging capacity of Hsp90

**Figure 4:**
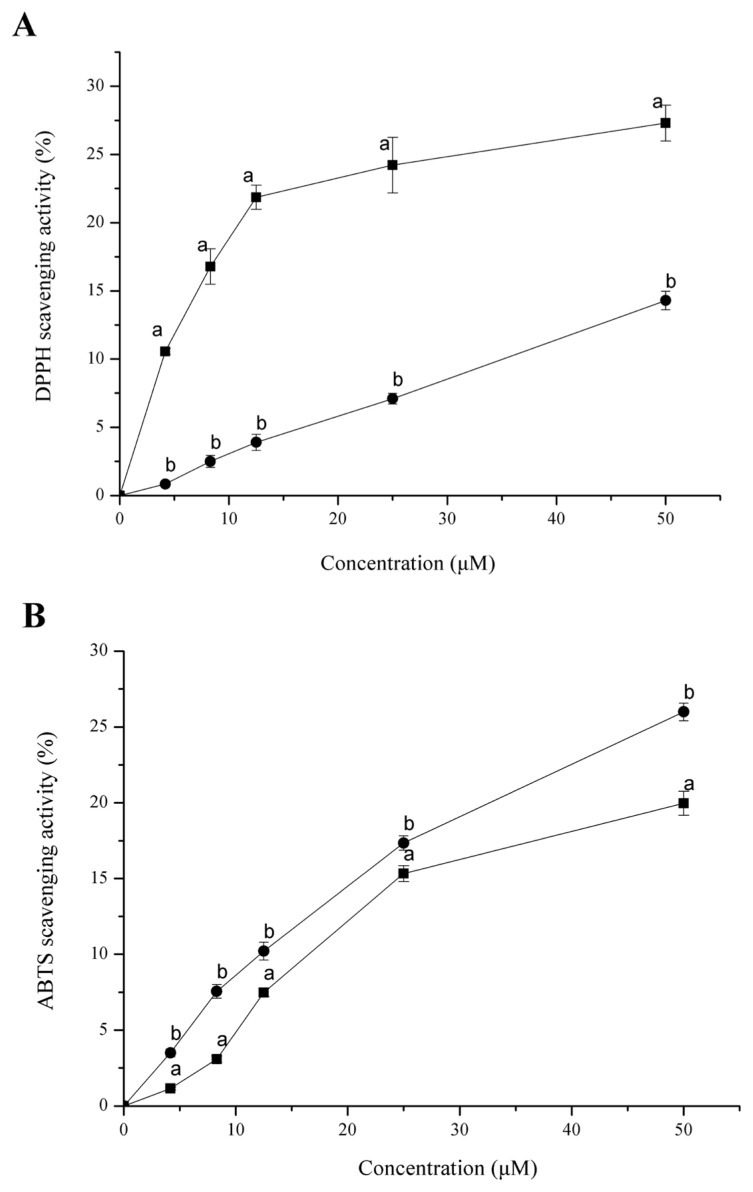
DPPH (A) and ABTS (B) radical scavenging activity of Hsp90 (■) and glutathione (●). DPPH and ABTS scavenging activity increased with the protein concentration. DPPH, 1,1-diphenyl-2-picrylhydrazyl); ABTS, 2,2′-azinobis(3-ethylbenzthiazoline-6-sulfonic acid; Hsp90, 90 Kda heat shock protein. Bars indicate standard deviation. ^a,b^ Different letters indicate significant difference (p<0.05).

With the increase of Hsp90 and GSH concentration, the DPPH and ABTS scavenging capacity gradually increased (Figure 4). DPPH is a stable free radical that shows maximal absorbance at 517 nm in ethanol. The DPPH assay is based mainly on the electron transfer reaction, while hydrogen-atom abstraction is a marginal reaction pathway [22]. When DPPH encounters a proton-donating substance (H^+^), the radical is scavenged by changing colour from purple to yellow and the absorbance is reduced. In our DPPH test, Hsp90 reduced the DPPH radical to a yellow-coloured compound, and the scavenging ability of protein can be up to 25% at the highest protein concentration. The DPPH radical scavenging ability of Hsp90 is significantly higher than that of the GSH (p<0.05). The ABTS method is a useful tool in determining the antioxidant activity of both lipopholic and hydrophilic antioxidants [23]. The scavenging activity against ABTS radical indicates the ability of protein to act as electron donors or hydrogen donors in free radical reactions [22]. The Hsp90 exhibited around 20% of ABTS scavenging activity at 50 μM, but it was significantly lower than GSH at the same concentration (p<0.05).

### Hydroxyl radical and PTIO· scavenging activity of Hsp90

**Figure 5:**
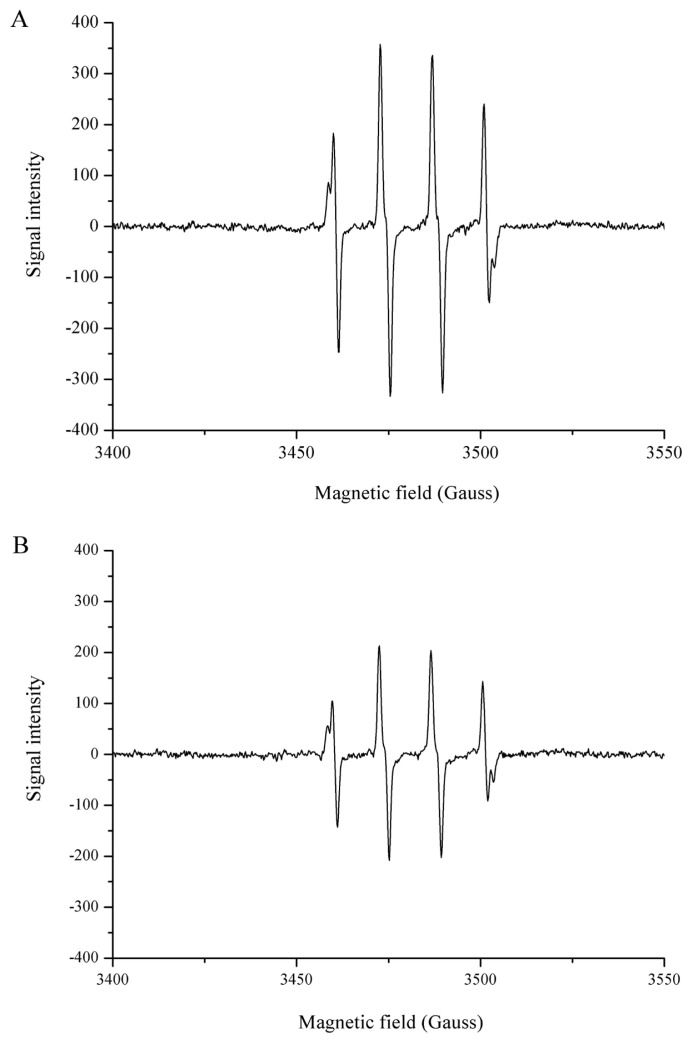
Electron paramagnetic resonance spectra of ·OH in sodium phosphate buffer (50 mM, pH 7.4) without (A) and with (B) Hsp90. Signal of ·OH decreased after the addition of 2.5 μM Hsp90. ·OH, hydroxyl radical; Hsp90, 90 Kda heat shock protein.

**Figure 6:**
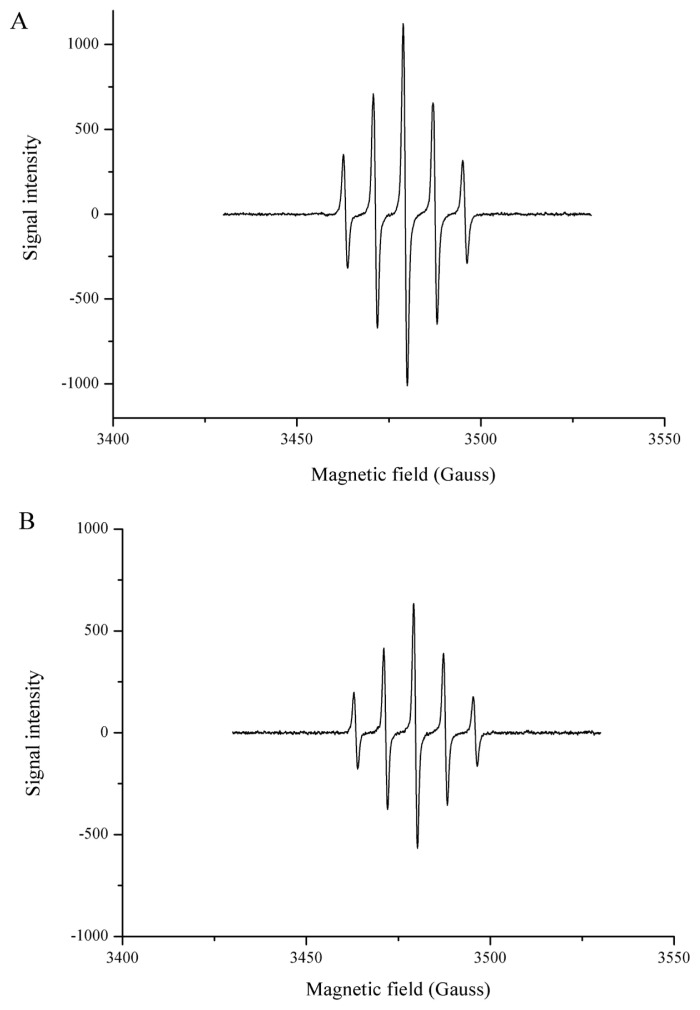
EPR spectra of ·PTIO in sodium phosphate buffer (50 mM, pH 7.4) without (A) and with (B) Hsp90. Signal of ·PTIO decreased after the addition of 2.5 μM Hsp90. Hsp90, 90 Kda heat shock protein; EPR, electron paramagnetic resonance; PTIO, 2-phenyl-4,4,5,5,-tetramethylimidazoline-1-oxyl-3-oxide.

DPPH and ABTS, which are widely used for *in vitro* antioxidant assays, are nitrogen-centered radicals [24]. Hence, the DPPH assay and the ABTS assay are preferred for “RNS scavenging” models and not “ROS scavenging” models [24]. Typical ROS forms are transient and have a very short half-life, and can be trapped only by EPR spectroscopy. EPR is the most specific and reliable, and has been used to directly detect the radical present in food or the antioxidant properties of food components [25,26]. As shown in Figure 5, a spectrum of 1:2:2:1 quartet was observed which is indicative of the BMPO-hydroxyl radical adduct (BMPO-OH). When Hsp90 was added to the system, the EPR signal intensity decreased (Figure 5b), indicating Hsp90 was effective in the scavenging of hydroxyl radicals. The hydroxyl radical is one of the major causes of oxidative damage, due to its high standard redox potential and its low selectivity, reacting indiscriminately with lipids, proteins and nucleic acids [27]. As seen in Figure 6, PTIO· presented a characteristic EPR signal of 1:2:3:2:1 quintet splitting, the presence of Hsp90 decreased the PTIO· signal intensity (Figure 6b). PTIO· is a stable radical that is usually used to detect nitric oxide. The structure of PTIO· shows the unpaired electron is located in the O atom, thus it is an oxygen-centered radical and can be applied to evaluate the ROS scavenging ability of the antioxidant, and the amine oxide zwitterion moiety makes it a hydrophilic species [24]. The direct ROS scavenging ability involves hydrogen atom transfer or proton transfer pathway, and the PTIO· scavenging has been demonstrated to be involved in at least hydrogen atom transfer [24,28].

## CONCLUSION

In the present study, the antioxidative ability of Hsp90 isolated from meat was assessed. Results showed Hsp90 could bind with oxidized phospholipid, and prevent their further oxidation to secondary products. Hsp90 could also scavenge DPPH and ABTS radicals, and had the ability to quench hydroxyl and PTIO· radicals. Our findings contribute to the understanding of direct antioxidative role of Hsp90, further characterization and screening of oxidized phospholipids and their binding proteins may be exciting area of research and have potential in the prevention of lipid oxidation and improvement of meat quality.

## Figures and Tables

**Table 1:** Binding of Hsp90 to various compositions of liposome as analyzed by surface plasmon resonance

Lipids	RU	Rmax	Rmax/RU	Ka (×104 M^−1^s^−1^)	Kd (×10^−3^s^−1^)	KD (×10^−7^ M)
PAPC	1,800±32^b^	1,324±32^a^	0.74±0.02^a^	2.55±0.18^b^	2.71±0.34^b^	1.06±0.06^a^
oxPAPC	1,700±15^a^	1,645±30^b^	0.97±0.01^b^	1.00±0.55^a^	1.68±0.20^a^	1.67±0.62^a^

Hsp90, heat shock protein; RU, resonance unit; Ka, association rate constant; Kd, dissociation rate constant; KD, equilibrium dissociation constant, calculated as the ratio of the Kd and Ka; PAPC, L-a-1-palmitoyl-2-arachidonoyl-sn-glycero-3-phosphorylcholine; oxPAPC, oxidized L-a-1-palmitoyl-2-arachidonoyl-sn-glycero-3-phosphorylcholine.

a,b Means are different at p<0.05.
